# Persistence and adherence to biologic therapies in juvenile idiopathic arthritis

**DOI:** 10.1038/s41598-021-95252-8

**Published:** 2021-08-10

**Authors:** Juan Carlos Nieto-González, Laura Trives-Folguera, Alejandra Melgarejo-Ortuño, Aranzazu Ais, Belén Serrano-Benavente, María Sanjurjo, José María Álvaro-Gracia, Indalecio Monteagudo Sáez

**Affiliations:** 1 Rheumatology Department, Hospital General Universitario Gregorio Marañón, Madrid, Spain; 2 Pharmacy Department, Hospital General Universitario Gregorio Marañón, Madrid, Spain

**Keywords:** Musculoskeletal system, Rheumatic diseases

## Abstract

Juvenile idiopathic arthritis (JIA) is a chronic inflammatory disease that often requires biological therapy to control its activity. Medication persistence and adherence are important aspects on which we have scarce information. We performed a longitudinal, retrospective, and observational study based on data from the daily clinical management of JIA patients. We recorded clinical remission at 6 and 12 months. Persistence of biological therapy was evaluated using Kaplan–Meier curves, and adherence was assessed using the medication possession ratio (MPR). We included 68 patients who received biological therapy. Of these, 11 (16.2%) and 5 (7.4%) required a second and third drug, respectively. The persistence rate for biological therapy at 5 years was 64%, with no differences between the first and second lines. Adherence was high during the first year of treatment (MPR80: 96.3%) and also in the second and third years (MPR80: 85.2% and 91.8%, respectively). Persistence and adherence to biological therapy were remarkably high in our JIA cohort. Adherence to biological treatments could be related to a higher probability of fulfilling the Wallace remission criteria at 6 months, although this was not confirmed at 12 months.

## Introduction

Juvenile idiopathic arthritis (JIA) is a heterogeneous disease that comprises all types of arthritis lasting longer than 6 weeks in patients under the age of 16 years^1^. Treatment should be adjusted to each JIA subcategory, although many patients need systemic drugs^2^. These include synthetic disease-modifying antirheumatic drugs (sDMARDs), mainly methotrexate^3^, and biological therapy (bDMARDs), mostly tumor necrosis factor alpha (TNFα) inhibitors^4,5^.

Polyarticular JIA requires bDMARDs in around 50% of cases to reach the treatment goal^6^, which is remission^7,8^. However, more important than choosing a specific treatment per se is the requirement to follow a treat-to-target (T2T) approach, which improves disease activity irrespective of the systemic drugs used^8–10^.

Adherence is the extent to which a patient acts in accordance with medical prescriptions or recommendations^11^. According to the World Health Organization (WHO), adherence stands at around 50% for chronic disease, both in adults and children, and plays a key role in the efficacy of treatment^11^. In children, adherence is difficult to measure owing to factors such as parental responsibility. However, in patients taking bDMARDs, adherence can be calculated using the medication possession ratio (MPR), which is based on the electronic dispensing record kept by the hospital pharmacy service.

Adherence has been sometimes evaluated by calculating medication persistence^12^, although these concepts differ. Persistence is the entire time that the patient continues to take the treatment and ends when the patient stops the treatment for any reason^13–17^. Compared with adult data on bDMARD persistence, many JIA patients stop their medication owing to clinical remission, thus potentially explaining differences in persistence^12–17^.

The aim of our study was to calculate adherence to bDMARDs in our JIA cohort during the first 3 years of treatment. We also aimed to calculate drug persistence for bDMARDs.

## Methods

We conducted a longitudinal, observational, and retrospective study of our JIA cohort. The study was carried out in accordance with relevant guidelines and regulations, and its design was in line with current regulations and with the approval of the Spanish Agency of Medicines and Medicinal Products. We included all JIA subcategories and all bDMARDs. We excluded patients who were treated with only steroids and/or sDMARDs and patients who were naïve for bDMARDs. We also excluded patients with less than 6 months of treatment from the adherence calculation. All patients and parents agreed to participate in the study, and informed consent was provided by the parents or patients when applicable. The study was approved by the local ethics committee (Comité de Ética de la Investigación con Medicamentos, Hospital General Universitario Gregorio Marañón, Madrid, Spain).

We collected clinical and laboratory data from the electronic medical records. We reviewed the start and end dates for biological therapy and the reasons for ending therapy. The main reasons for ending a bDMARD were reaching remission, appearance of adverse events, patient or parental request, and financial aspects. We also reviewed clinical remission at 6 and 12 months after initiation of a biological treatment.

The MPR was calculated based on data from our pharmacy registry of biologic therapy obtained from electronic prescriptions as the number of doses dispensed in relation to the dispensing period. It is usually expressed as a percentage. We calculated the annual adherence to the first line of biologics during the first 3 years of treatment.

For practical and statistical reasons and based on pathophysiology, we created 4 homogeneous groups of JIA; group 1, oligoarticular JIA (including persistent and extended subcategories); group 2, polyarticular JIA (including rheumatoid factor–positive and –negative subcategories); group 3, juvenile spondyloarthritis, (including enthesitis-related arthritis and psoriatic arthritis); and group 4, systemic JIA. We used these groups to compare persistence and clinical data between the different subcategories.

## Definitions

> *Lack of adherence*: Lack of adherence to bDMARDs was defined as an MPR lower than 80% per year (MPR < 80)^18^. We also applied a more exacting criterion by defining lack of adherence as an MPR lower than 90% per year (MPR < 90).

An adherence of 80% or higher is represented as MPR80 and MPR90 for adherence of 90% of higher.

> *Clinical remission*: Patient fulfilling the Wallace criteria with or without treatment. No arthritis, enthesitis, dactylitis, uveitis, or systemic features, such as fever.

## Statistics

Data for continuous variables are expressed as mean and standard deviation (SD) or median and interquartile range (IQR). Data for qualitative variables are expressed as frequencies and percentages. Biological persistence was studied using Kaplan–Meier curves. Comparisons between groups were made using the Pearson chi-square test, *t* test, or Mann Whitney test, as applicable. All statistical tests were performed 2-sided, and a p-value of < 0.05 was considered statistically significant. All calculations were performed using IBM SPSS Statistics for Windows, Version 25.0 (Armonk, NY: IBM Corp).

## Results

From a total of 132 JIA patients followed in our unit, we included 68 patients (51.5%) who had received 1 or more bDMARDs. Of these, 11 (16.2%) needed a second bDMARD and 5 (7.4%) a third bDMARD. Therefore, in total we analyzed 84 exposures to biologics: 72 (85.7%) with TNFα inhibitors, 8 (9.5%) with interleukin-6 inhibitors, and 4 (4.8%) with other bDMARDs. Table 1 summarizes the demographic data for the study population according to whether or not they had reached remission at 12 months.

**Table 1:** Demographic data of patients with juvenile idiopathic arthritis and comparison between groups by remission at 12 months with their first biologic therapy.

	All patients n: 68	Remission at 12 months n:58	Not remission at 12 months n:10	*p*
Female sex, n (%)	39 (57.4%)	36 (94.7%)	2 (5.3%)	0.013
Age, mean (SD)	6.6 (4.7)	6.6 (4.5)	8.9 (4.7)	0.192
**JIA subcategory**				
Systemic	5 (7.4%)	5 (100%)	0	0.508
Oligoarticular	29 (42.6%)	22 (84.6%)	4 (15.4%)	
Polyarticular	19 (27.9%)	16 (88.9%)	2 (11.1%)	
Enthesitis-related	11 (16.2%)	15 (88.2%)	2 (11.8%)	
Psoriatic	4 (5.9%)			
ANA-positive	27 (39.7%)	21 (87.5%)	3 (12.5%)	0.704
Chronic anterior uveitis	7 (10.3%)	6 (100%)	0	0.224
1st biologic	68 (100%)	41 (85.4%)	7 (14.6%)	0.970
2nd biologic	11 (16.2%)	9 (81.8%)	2 (18.2%)	
3rd biologic	5 (7.4%)	4 (80%)	1 (20%)	

JIA, juvenile idiopathic arthritis; MPR, medication possession ratio; ANA, antinuclear antibodies; SD, standard deviation.

The most common bDMARD was etanercept, which was taken by 52 patients (76.5%), followed by adalimumab in 9 patients (13.2%), tocilizumab in 3 patients (4.4%), anakinra in 2 patients (2.9%), and infliximab and ustekinumab in 1 patient (1.5%) each. The mean time to starting a bDMARD was 1.1 years (interquartile range [IQR] 0.3–3.2), and the mean time to tapering of the first biologic was 0.9 years (IQR 0.5–1.2). We found that female patients had a higher probability of achieving remission at 12 months than male patients (p:0.013), with no other differences for the JIA subcategory (p:0.617), uveitis (p:0.224), or antinuclear antibody (ANA) status (p:0.704).

### Persistence of biologic therapy

**Figure 1:**
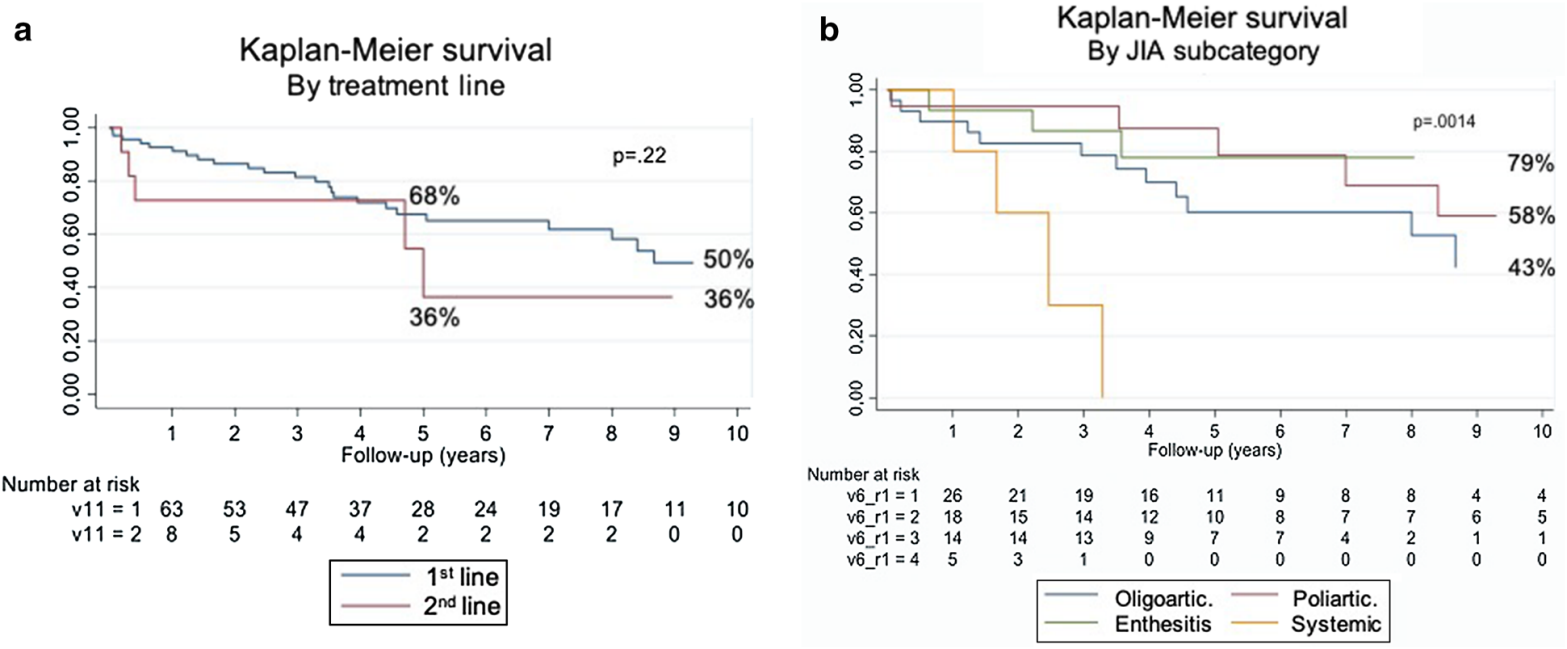
Kaplan–Meier curves for persistence of biologics by line of treatment (**a**) and by JIA subcategory (**b**).

We calculated the overall persistence of biologic therapy at 5 years and compared the persistence of first- and second-line agents over a 10-year follow-up period. The overall persistence after 5 years of follow-up was 64%. There were no differences in persistence between the first and second lines of biologic treatment. However, persistence of biologics in the systemic JIA (group 4) was significantly shorter, with no differences between the rest of the JIA subcategories. The Kaplan–Meier curves for the first- and second-line biologics and for the different JIA subcategories are shown in Fig. 1.

The reasons for discontinuation of biologics at any time (31 patients, 36.9%), were remission in 15 cases (48.4%), inefficacy in 9 cases (29%), adverse events in 4 cases (12.9%), and other causes in 3 cases (9.7%). The mean time of bDMARD treatment before stopping medication (15 cases) due to remission was 4.1 years, and all but 2 cases continued with methotrexate as a systemic treatment after stopping bDMARDs.

### Adherence to biologic therapy

**Figure 2:**
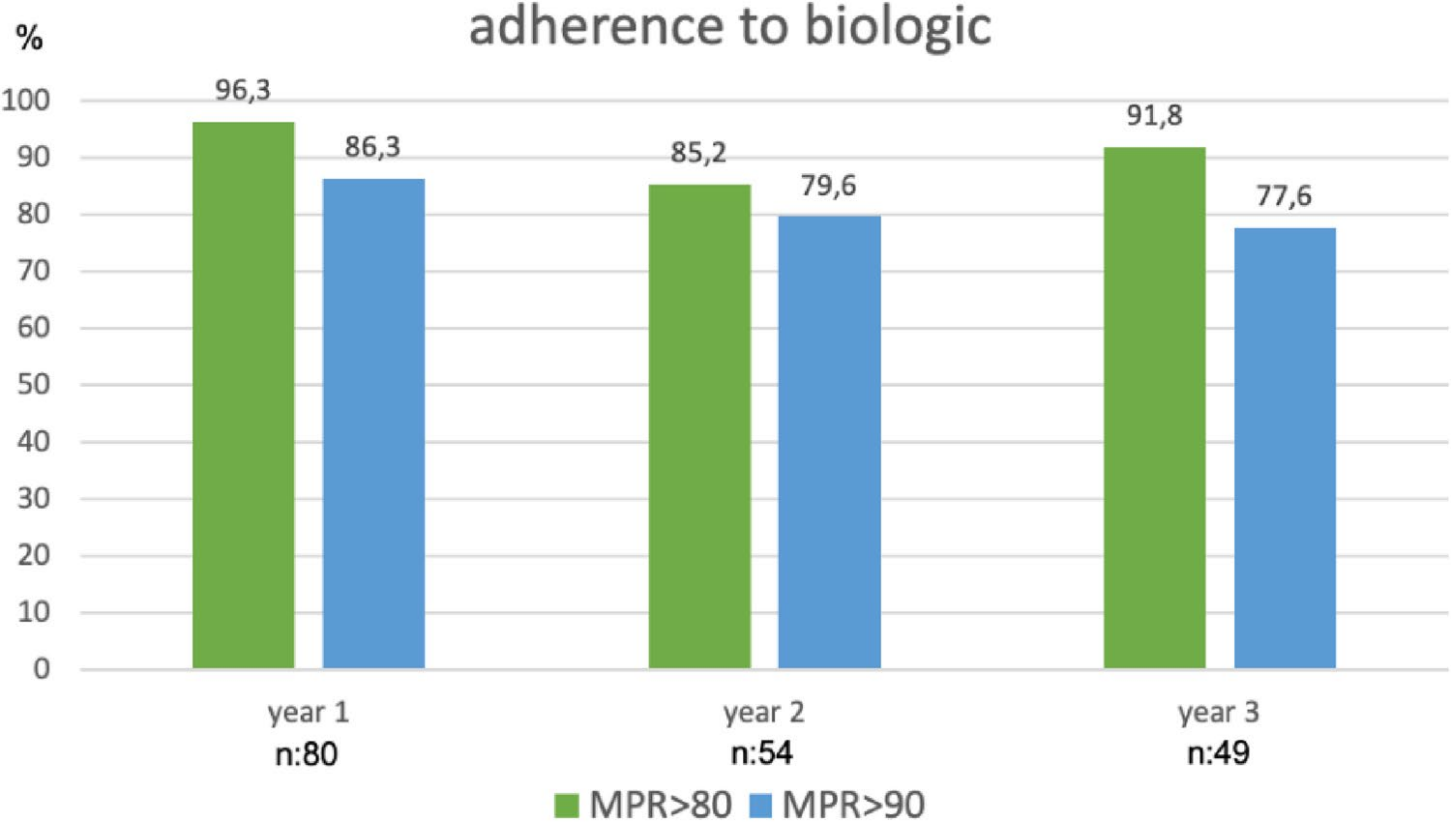
Adherence to therapy with a biologic during the first 3 years of treatment with a medication possession ratio (MPR) greater than 80% and 90%.

Adherence to bDMARDs at 1 year was 96.3% with MPR80 and 86.3% with MPR90. The percentages of adherence in patients who continued the treatment were similar after 2 and 3 years, although they decreased slightly over time. Figure 2 shows adherence over 3 years of therapy with a biologic. There were no differences between adherent and non-adherent patients, irrespective of whether they were divided by JIA subcategory (p:0.972), sex (p:0.398), or ANA status (p:0.217).

We evaluated whether adherence to the biologic had any influence on the possibility of achieving clinical remission at 6 and 12 months (Table 2). When we selected MPR80, statistically significant differences were observed for remission at 6 months (p:0.039) but not at 12 months (p:0.153). Moreover, these differences were not observed when we selected MPR90 (p:0.144 and 0.274 respectively).

**Table 2:** Remission (Wallace criteria) at 6 and 12 months after initiation of biologic therapy with respect to adherence to treatment during the first year.

	Remission at 6 months (n:80)				Remission at 12 months (n:68)		
	Yes (n:64)	No (n:16)	p		Yes (n:58)	No (n:10)	p
**1st year (n:80)**							
MPR80 (n:77)	63 (81.8%)	14 (18%)	**0.039**	MPR80 (n:66)	57 (86.4%)	9 (13.6%)	0.153
MPR < 80 (n:3)	1 (33%)	2 (66.7%)		MPR < 80 (n:2)	1 (50%)	1 (50%)	
MPR90 (n:69)	57 (82.6%)	12 (17.4%)	0.144	MPR90 (n:61)	53 (86.9%)	8 (13.1%)	0.274
MPR < 90 (n:11)	7 (63.6%)	4 (36.4%)		MPR < 90 (n:7)	5 (71.4%)	2 (28.6%)	

In bold are showed the group differences statistically significant.

## Discussion

We usually follow a T2T strategy in the management of JIA, exactly as we do in adult patients with chronic arthritis. Our study showed high remission rates, persistence, and adherence to biological therapy in JIA. This T2T strategy, highly recommended by international task forces^10^, might be the reason for such favorable results. We found a remission to be more frequent in female than in male patients; however, this is not easy to explain, because sex has not been reported to affect disease severity, treatment response, or adherence.

Around 50% of the patients needed biologic therapy to control disease activity, and 16% and 7.6% needed a second and third biologic, respectively. Our results are similar to those of international studies in which the authors also followed a T2T strategy^6,8,9^. However, the clinical response in our cohort was slightly better, with complete remission after 6 months in more than three quarters of the patients, increasing to more than 80% at 12 months. A recent study showed 48% of patients to be in remission at month 12 after following a T2T strategy^8^, although the authors reported that 37% of patients were taking bDMARDs compared with 51.5% in our study, thus explaining in part the differences in findings. Moreover, a systematic literature review estimated that up to 68% of patients reached remission after 12 months of treatment^19^.

### Persistence of biological therapy

Follow-up was long for most of the patients, and we were able to calculate the persistence of biologics until 10 years of follow-up in some cases. Persistence at 5 years was remarkably high, with numerical but not statistical differences between the first and second lines of treatment. One study reported persistence of around 50% at 1 year^18^, and another study reported a higher persistence of 50–60% for TNFα inhibitors at 5 years^12^. Our results were higher and similar to those of a German cohort^12^ (89.9% at 1 year and 64% at 5 years). It is also very interesting that approximately 50% of patients discontinued biologic treatment due to remission, although elsewhere, only around 9–13% of JIA patients discontinued TNFα inhibitors due to remission^12,14,16^.

### Adherence to biological therapy

Adherence to therapy is essential if the clinical response is to be good^11^. The WHO estimated adherence to chronic treatments to be approximately 50%^11^ in adults and children. Previous studies reported maximum adherence (MPR80) to TNFα inhibitors of 42.1%^17^, and a recent literature review of adherence to treatment in JIA showed a maximum adherence of 65%^19^. However, results were higher (MPR80 96.3%) in our JIA cohort, which could be partially explained by the important efforts made by our Pharmacy Department during follow-up to optimize therapy with bDMARDs. Adherence can be limited by numerous barriers (eg, the patient, the patient’s family, and the kind of drugs^20^), which must be taken into consideration if we are to improve it.

Since we had remarkably high adherence results, we applied more exacting criteria by using MPR90. The proportion of adherent patients was still higher than in previous reports at 1 year (MPR90: 86.3% vs. MPR80: 38.4%)^17^. Moreover, the high adherence to biologic treatment was also maintained in our cohort during the second and third years.

Based on the Wallace remission criteria at 6 and 12 months, MPR < 80 was related to a reduced probability of achieving a remission at 6 months, although this was not confirmed at 12 months, probably owing to the small number of patients with active disease at this time. At 6 months 3 out of 77 patients (3.89%) had a MPR < 80 and the proportion was even smaller at 12 months, 2 out of 66 patients (3%).

### Limitations of our study

The major limitation of our study is the small sample size in a very heterogeneous disease such as JIA. It would be interesting to analyze each subcategory separately and compare them with each other. To do so, it is imperative that collaborative studies be designed to ensure sufficient patients in each JIA subcategory. Our study is also limited by its observational and retrospective study design, although this is the usual approach when studying persistence of and adherence to treatment.

## Conclusions

Persistence of and adherence to biologic treatment in JIA were very good in our cohort. While the reasons for discontinuing biologic treatment vary, we found remission to be a frequent motive. Adherence to biologic treatments could be related to a higher probability of fulfilling the Wallace remission criteria at 6 months; however, this was not confirmed at 12 months.
